# Separating sexual dimorphism from other morphological variation in a specimen complex of fossil marine reptiles (Reptilia, Ichthyosauriformes, *Chaohusaurus*)

**DOI:** 10.1038/s41598-018-33302-4

**Published:** 2018-10-08

**Authors:** Ryosuke Motani, Jiandong Huang, Da-yong Jiang, Andrea Tintori, Olivier Rieppel, Hailu You, Yuan-chao Hu, Rong Zhang

**Affiliations:** 1 Department of Earth and Planetary Sciences, University of California, Davis, CA 95616-8605 USA; 2 Department of Research, Anhui Geological Museum, Jiahe Road 999, Hefei, Anhui 230031 People’s Republic of China; 3 Laboratory of Orogenic Belt and Crustal Evolution, Ministry of Education, Department of Geology and Geological Museum, Peking University, Beijing, 100871 People’s Republic of China; 4 Dipartimento di Scienze della Terra, Università degli Studi di Milano, Via Mangiagalli 34-20133, Milano, Italy; 5 Integrative Research Center, The Field Museum, Chicago, IL 60605-2496 USA; 6 Institute of Vertebrate Paleontology and Paleoanthropology, Chinese Academy of Science, 142 Xizhimenwai Street, 100044 Beijing, China

## Abstract

The Early Triassic Chaohu Fauna from Anhui Province, China, contains the oldest record of Mesozoic marine reptiles, such as *Cartorhynchus* and *Sclerocormus*. Most specimens from the fauna belong to the ichthyosauriform *Chaohusaurus*, more specifically resembling *C*. *chaoxianensis*. However, a wide range of morphological variation exists within about 40 skeletons that have been prepared, likely reflecting mixed signals from both sexual and taxonomic differences. We test whether the sexual and taxonomic signals are separable based on quantification, aided by the knowledge of sexual dimorphism in extant marine tetrapods. There are two different suites of dimorphism that divide the specimens differently from each other yet consistently within each suite, resulting in four morphotypes in combination, likely representing two sexes of two taxa. Presumed males have larger ‘organ of prehension’ sensu Darwin, specifically limbs in the present case, for a given body length. This sexing criterion is supported by the only specimen of a gravid female, which belongs to the morphotype with short limbs. Males also have larger skulls for the trunk length compared to females. This study demonstrates that sexual and taxonomic signals are separable in fossil reptiles, with a sufficient sample size and careful analyses.

## Introduction

Sexual dimorphism is a biological phenomenon that may confuse the taxonomy of fossil species. There are three realms of morphology that differ depending on sex, namely primary sexual, secondary sexual, and non-reproductive characters^1–3^. Primary sexual characters—reproductive organs—are expected to be sexually dimorphic by default. Secondary sexual characters, i.e., features apart from the reproductive organs that aid reproductive success, are also expected to be sexually dimorphic. Finally, features that are not directly related to reproduction may also be dimorphic depending on sex, e.g., females and males may have different ecology-related morphologies because they live in different places or eat different food^1–3^.

It is usually difficult to sex fossil specimens with confidence unless the primary sexual character is preserved^4,5^. However, in case of fossil reptiles, it is very unlikely that their reproductive organs are preserved except in rare cases where these organs are associated with mineralized hard tissues. The presence of eggs or embryos in the trunk of an adult individual may indicate that the individual is female, yet not all females are expected to be pregnant upon fossilization. This makes some secondary sexual characters the most useful for sexing fossil reptiles. Of the secondary sexual characters, sexual size dimorphism, which is used in many ecological studies^6^, is usually unknown until sexing is done first based on criteria other than size. It is therefore not very useful in sexing of fossil forms^5^. Sexual shape dimorphism, on the other hand, is useful in sexing fossil reptiles but it requires sufficiently large sample sizes^3,7^ that cover a range of growth stages because growth rates of secondary sexual characters tend to accelerate or decelerate during post-embryonic ontogeny^5^. Given that not all shape dimorphisms are primary or secondary sexual characteristics, it is often very difficult to decide whether differences in ecologically relevant characters represent sexual or taxonomic distinctions in the absence of information from clearly primary or secondary sexual characters.

Another factor that may further complicate sexing of fossil reptiles is taxonomic variation. Given that extinct reptiles are known only from fossilized bones in most cases, it is very difficult to distinguish two distinct species that resemble each other in a mixed collection of specimens, when they may also be subject to slightly different degrees of sexual dimorphism. Such a heterogeneous collection of specimens may reveal a confusing mixture of taxonomic variation and sexual dimorphism. For example, sexually dimorphic characters in a species may exhibit a bimodal distribution of shape variation, yet the bimodality may disappear when two taxa are mixed. Thus, unless the taxa are identified first, it is difficult to decipher sexual dimorphism, and vice versa. In such a case, a comprehensive approach is necessary that simultaneously considers both sexual dimorphism and taxonomic variation.

Repeated field excavations were held at Majiashan in Chaohu, Anhui Province, China by a joint research team by Anhui Geological Museum, the Peking University, University of California, Davis and University of Milan, starting in 2010. These efforts resulted in the collection of more than 60 vertebrate skeletons from the middle to late Spathian, Early Triassic. About 40 specimens of the better-preserved marine reptiles from this collection have been prepared, enabling for the first time a detailed study of morphological evolution in Early Triassic ichthyosauriforms from a single locality based on a large sample size. Most of the specimens belong to the genus *Chaohusaurus*, which is a basal ichthyosauriform. Two species of *Chaohusaurus* have been described from the region, namely the type species *C*. *geishanensis* and a referred species *C*. *chaoxianensis*. The distinction between the two species was once considered obscure when the sample size was small^8^ but a recent revision clarified that they are distinct from each other^9^. Most notably, *C*. *geishanensis* has a short carpus with densely set elements, while *C*. *chaoxianensis* has a longer carpus with poorly ossified elements. Other ichthyosauriforms from the locality are *Sclerocormus*^10^ and *Cartorhynchus*^11^.

Most of the newly prepared skeletons resemble *Chaohusaurus chaoxianensis* in that their mesopodia are poorly ossified and elongated^12^ compared to those of the type species *C*. *geishanensis*^13^. We tentatively refer to this collection of the specimens as the *C*. *chaoxianensis* complex. However, much morphological variation across the body is present among the specimens in this complex. For example, the degree of carpus elongation is variable^12^ while some specimens are evidently longer-snouted than others. Some specimens have branched neural spines near the caudal peak in the tail when others do not, and limbs are longer relative to the body in some specimens than in others. Confusingly, these dichotomous characters do not congruently sort the specimen complex into groups. The observed incongruence suggests that some of the characters may represent sexual shape dimorphisms while some others reflect taxonomic variation, and the rest may be due to simple individual variation. Given that the sample size can now be considered sufficiently large for a fossil reptile, it may be possible to clarify the taxonomy, sexual shape dimorphism, and individual variation in this specimen complex.

The purpose of the present study is to identify sexual dimorphism, taxonomic variation, and individual variation in the *Chaohusaurus chaoxianensis* complex, based on both qualitative and quantitative characters. To judge if a given set of dimorphic characters in the *C*. *chaoxianensis* complex represents sexual dimorphism, it is useful to know the distribution of sexual dimorphism in extant marine tetrapods, especially cetaceans, because features related to reproductive success in water are expected to be secondary sexual. We therefore start by reviewing the known sexual dimorphism in extant aquatic tetrapods.

## Sexual Dimorphism in Extant Aquatic Tetrapods

Much of the sexual dimorphism known in extant aquatic tetrapods is sexual size dimorphism (SSD), as evident in pinnipeds^14^, cetaceans^14^, sea kraits^15^, and sea snakes^16^, although sea turtles exhibit little SSD^17^ unlike more terrestrial turtles that tend to have female-biased SSD^18,19^. Unfortunately, as stated earlier, it is difficult to assess SSD based on fossils unless the specimens are first sexed based on criteria other than the body size^5^. We therefore focus on sexual shape dimorphism.

A review of the literature on aquatic tetrapods suggests that the male organ used to hold females during copulation tend to be larger than those of females of the same body size. These organs were called “organs of prehension” by Darwin^1,2^, who struggled to decide whether they were primary or secondary sexual. An organ of prehension may be elongated claws or a relatively long tail ending with a nail as in some turtles^17^, but more often it is the limbs, including those that turned into flippers. In cetaceans, sexual dimorphism often affects flipper length^14,20^, width^21^, or phalangeal count^22^. Similar sexual dimorphism in limb length is known in semiaquatic tetrapods such as some salamanders that engage in amplexus under water during copulation^23,24^, and has also been suggested for several pachypleurosaurs, such as *Dactylosaurus*, *Keichousaurus*, *Neusticosaurus*, and *Serpianosaurus* from the Triassic^5,25–27^. Not all cetaceans exhibit sexual dimorphism in flipper length^28^ but we did not find any case where females had longer flippers than males for a given body size.

Apart from the organs of prehension, a common sexual shape dimorphism in cetaceans is seen in the size of the pelvic bones relative to the body, which are larger in males than in females, likely reflecting the sexual differences in genital morphology^29,30^. Also, various cranial measurements are known to differ between males and females of some cetaceans^31–35^, including one case where the males typically have longer skulls than females^36^ as well as an opposite case where female skulls are longer than the male counterpart^37^.

## Results

### Qualitative features

We observed three discrete osteological features that vary within the sample. The first is the shape of the neural spines near the caudal peak in the tail, where the weak dorso-ventral curving of the tail vertebrae reaches the peak and the anticlination of the neural spines starts. In some specimens, one or two neural spines in the region are distinctly bifurcated into dorsal and anterior branches, while the bifurcation is absent or obscure in others. Even in the non-bifurcated type, there are two thickened axes within the neural spine that extends dorsally and anteriorly, respectively, corresponding to what appear as two branches in the bifurcated type. However, in the non-bifurcated type, the area in-between the thickened axes is bridged by a thin bony flange whose antero-dorsal margin is slightly concave but not strongly notched. This flange is extremely reduced or absent in the bifurcated type. The thinned area may be damaged in some specimens but such breakage is usually distinguishable from the naturally bifurcated morphology through careful observation (e.g., in AGBAGB7409 and AGBAGB7413).

**Figure 1:**
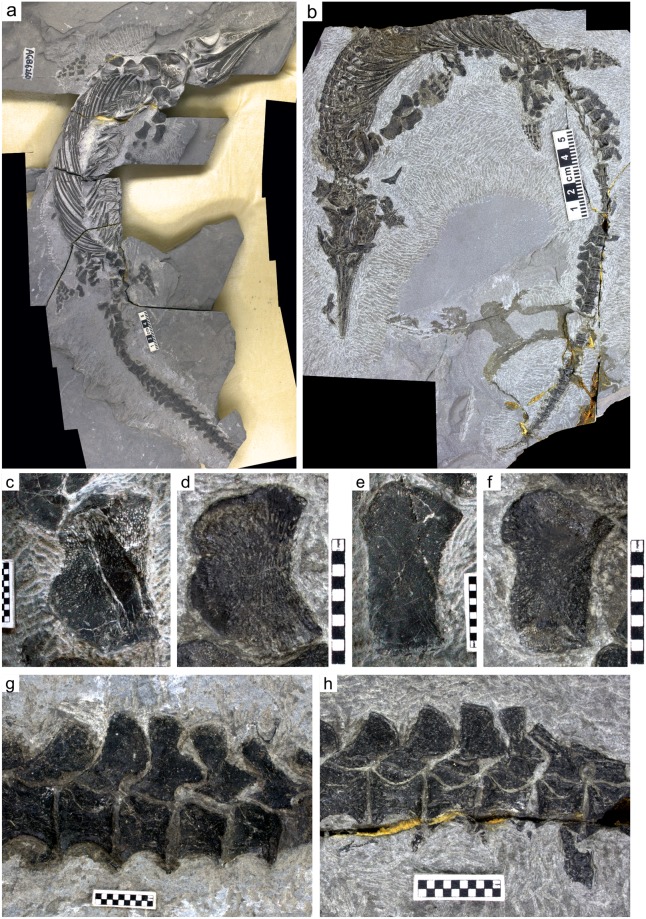
Qualitative characters to distinguish between the two proposed morphotypes. (**a**) AGB6260, A large individual belonging to Type B. (**b**) AGM-GB6262, a medium-sized individual of Type A. (**c**) Poorly-developed anterior flange of the humerus (Type B) with a wide and smooth notch in (**a**). (**d**) Well-developed anterior flange of the humerus (Type A) with a notch ending with a pointed tip in (**b**). (**e**) Radius with poorly developed anterior flange (Type B) in (**a**) laterally inverted. (**f**) Radius with a well-developed anterior flange with rounded antero-distal corner in (**b**). (**g**) Anticlined neural spines of the caudal peak region with bifurcation, due to poor development of a bony flange in (**a**). (**h**) Same without bifurcation thanks to the bony flange that fills the gap in (**b**). Scale bars are 5 cm long in (**a** and **b**), and made of 1-mm squares in (**c**–**h**).

The second is the antero-proximal flange of the radius, which is a unique feature of *Chaohusaurus chaoxianensis*. This flange is well-developed distally, revealing radial surface striations, in some specimens but poorly-developed in the others. In the well-developed type, the antero-distal margin of the flange is well-rounded thanks to the development of the flange (Fig. 1d), whereas the flange and shaft appear almost confluent with each other in the poorly-developed type (Fig. 1c). There seem to be some ontogenetic changes in the degree of the development of the flanges.

The third is the relative development of the anterior flange of the humerus, most evidently seen in the shape of the humeral notch in mature individuals, which may appear to end with a pointed corner (Fig. 1b), or with a widely open and smooth concavity (Fig. 1a). The anterior flange of *Chaohusaurus* humerus is a combination of the proximal and distal sub-flanges that develop from the proximal and distal ends. The gap between the two sub-flanges is referred to here as the notch. The notch is wide when the proximal and distally sub-flanges are poorly-developed, and the deepest part of the notch is widely open and smoothly curved. When the proximal and distal flanges are well-developed, however, the deepest part of the notch is very narrow and appears almost pointed. This humeral character is usually useless in recognizing morphotypes among young individuals, which tend to have the widely-open morphology along the humeral anterior margin because the anterior flange has yet to be fully developed. As with the neural spine flange, the anterior margin of the humerus may be damaged through over-preparation in some specimens (e.g., in AGB7413).

These three characters covary—those with an unbranched first robust anticline neural spine also have a well-developed radial flange and sharp notch along the anterior margin of the humerus (called Type A hereafter; Fig. 1b,d,f), while those with a bifurcated first anticline neural spine have a small radial flange and humeral notch that is wide and smooth (Type B hereafter; Fig. 1a,c,e). Note that all three characters concern the degree of development of bony flanges, along the anterior humeral margin, antero-proximal radial margin, and in-between the two thickened axes of the first robust anticline neural spine in the tail, respectively. Character states observed in Type A are all derived from enhanced development of the respective flanges while those in Type B represent reduction. Consequently, the congruence between the characters likely reflects a common developmental cause rather than being a mere coincidence.

As the three characters are all based on the relative development of flanges, there may be a concern that ontogeny may obscure their distinction to some extent. However, the feature of the neural spine is recognized regardless of size. The features of the humerus and radius vary with growth to some extent, but they are difficult to distinguish only in the smallest specimens. There are four specimens that are very small, namely AGBAGB6254, AGBAGB7411, AGB2906, and GMPKU P1101, whose trunk lengths are less than 170 mm, in contrast to 469 mm in the largest individual examined. The qualitative characters of the humerus and radius in these specimens are weakly expressed due to young age, sometimes making it difficult to identify them as either Type A or B with confidence based on the two forelimb features alone, while the tail is preserved only in AGB7411. They were tentatively assigned to the types that they resemble best, respectively. These initial assignments were all supported by additional considerations as discussed below. The next smallest specimen, AGB7409 with a trunk length of 174 mm, has a well-developed radial flange, while the humeral flange is not yet completely developed. This specimen has a breakage caused by an air chisel in an anticlination neural spine, making it appear as if it were bifurcated, but it belongs to Type A.

### Quantitative features

**Figure 2:**
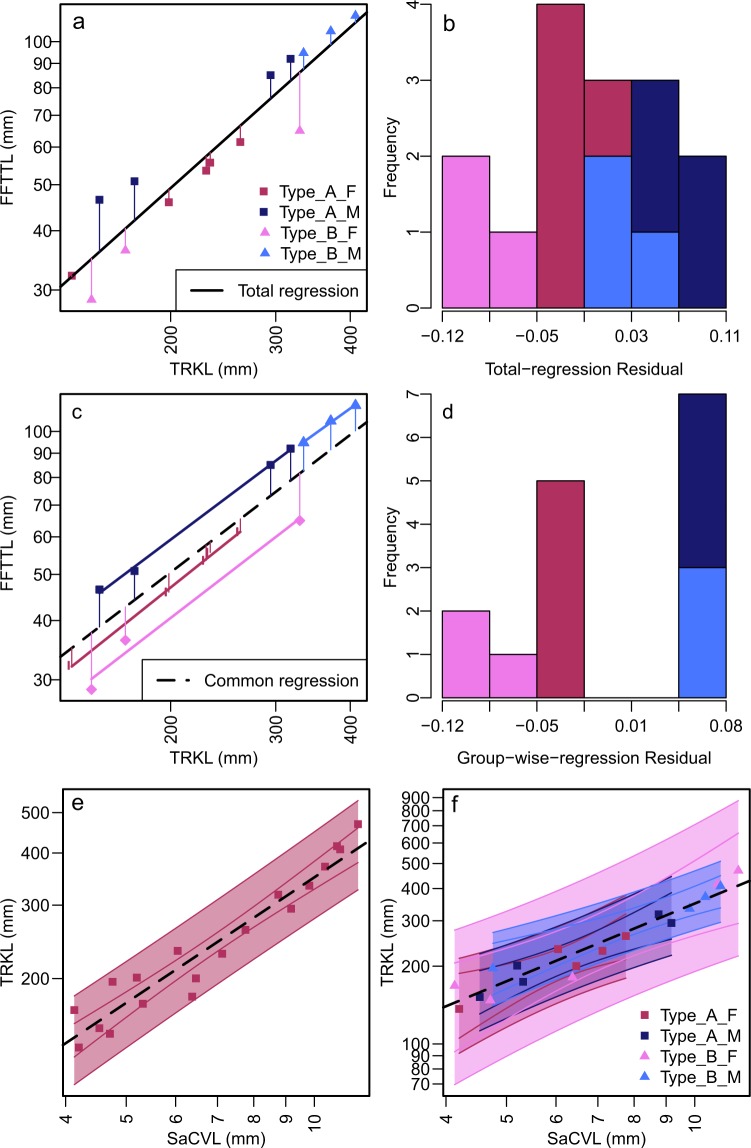
Bias from regression lines on unimodality of residual distribution and correlation between TRKL and SaCVL. (**a**) Regression of FFTTL on TRKL when all specimens are pooled. (**b**) Histogram of residuals from (**b**). (**c**) Regression of FFTTL on TRKL based on common regression lines among four morphotypes (dotted line). (**d**) Histogram of residuals from (**c**). Vertical legs from data points to respective regression lines are residuals. The use of common regression illuminates bimodal distribution of the residuals. (**e**) Regression of TRKL on SaCVL based on all specimens, revealing a wide prediction interval. (**f**) Regression of TRKL on SaCVL based on four morphotypes established in this study.

**Figure 3:**
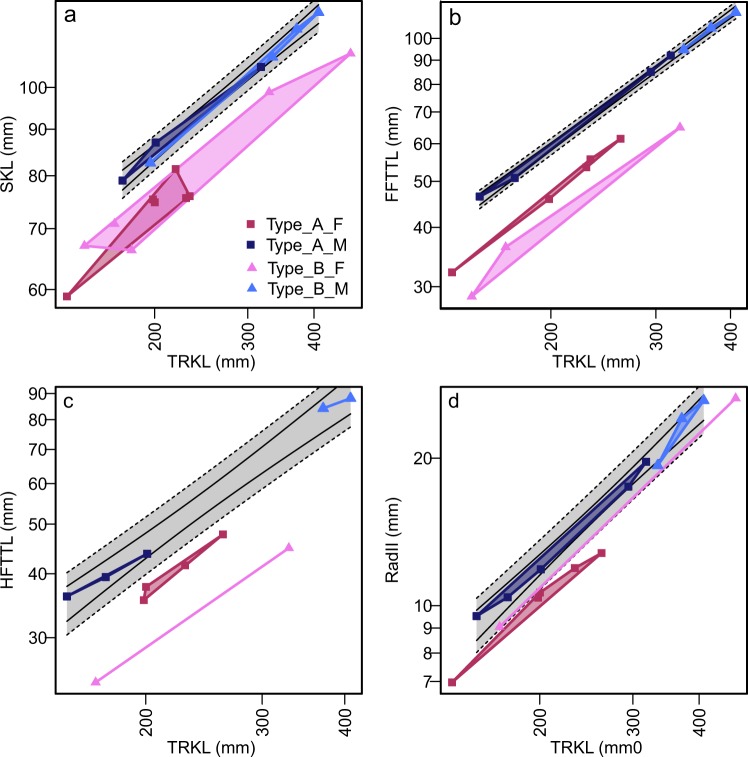
Sexual dimorphism in bone dimensions relative to trunk length. (**a**) SKL against TRKL. (**b**) FFTTL against TRKL. (**c**) HFTTL against TRKL. (**d**) RadII against TRKL. Colors indicate the four morphotypes established in the present paper. Black curves and gray areas are 95% confidence (solid line) and prediction (broken lines) intervals for pooled males.

The test of unimodality versus multimodality using all data found that none of the characters exhibited a clearly multimodal residual distribution, except perhaps the total length of the hind limb (p = 0.181, n = 11), while many characters revealed p-values higher than 0.9 or even 0.95, suggesting approximate unimodality (Fig. 2a,b). Once the samples were divided into Types A and B according to the qualitative characters, however, signals for multimodality became stronger within each morphotype (Fig. 2c,d). Two of the features with strong multimodality signals were congruent with each other in the way they sort the samples, namely the lengths of the forelimb (Fig. 3b) and hind limb (Fig. 3c). We used these quantitative features to divide samples into Subtypes 1 and 2, with Subtype 2 having longer flippers than Subtype 1 for a given trunk length. Importantly, these two subtypes are clearly separated from each other, without an intermediate form to fill the gap (Figs 2c,d and 3b,c). ANCOVA also supports the clear distinction between Subtypes 1 and 2 in the relative flipper lengths to the trunk length, with the following statistics: p = 0.0488, F = 4.81, n = 15 for the forelimb flipper, and p = 0.0387, F = 6.11, n = 11 for the hindlimb flipper (note that the sample size is smaller than in most other cases in the latter).

Based on the common tendency observed among extant aquatic tetrapods, individuals with longer forelimb (Subtype 2) are here interpreted as males and Subtype 1 as females (see Discussion). The total length of the skull also exhibits similar divisions between males and females, where males have longer skulls for a given trunk length (Fig. 3a). ANCOVA suggests that the differences between the male and female regression lines to be significant at p < 0.001 (F = 194.2, n = 18) for the skull length relative to the trunk length. The length of the carpus, measured as the distance between the radius and the second metacarpal^9^, also exhibited a similar division of the samples (Fig. 3d). ANCOVA again suggests a significant difference between males and females (p < 0.001, F = 240, n = 15).

**Figure 4:**
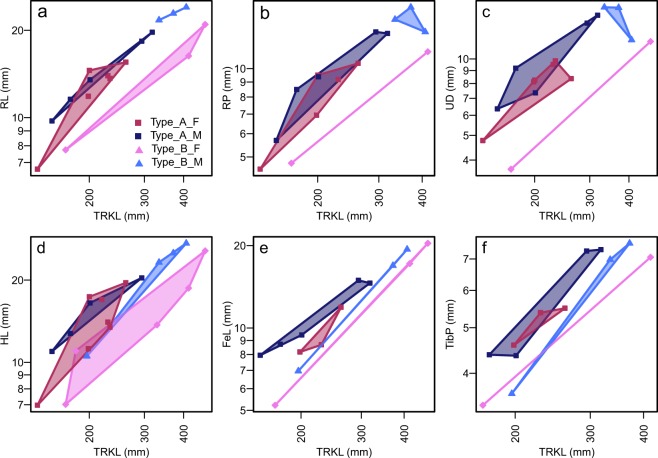
Features unique to females of Type B, and those unique to Type B. (**a**) RL against TRKL. (**b**) RP against TRKL. (**c**) UD against TRKL. (**d**) HL against TRKL. (**e**) FeL against TRKL. (**f**) TibP against TRKL. (**a**–**c**) Are unique to females of Type B. (**d**–**f**) Roughly divides Types A and B.

Some quantitative characters of the propodial and epipodial elements, especially concerning the hind limb, add to the morphological differences between Types A and B. The best example is the relative length of the femur to body trunk (Fig. 4e), where Type A tends to have longer femora than Type B for a given body trunk length. The difference is statistically significant (p = 0.007, F = 10.9, n = 14) according to ANCOVA. The relative length of the humerus to the trunk (Fig. 4d) also exhibits the same tendency, but the signal is not as strong as in the case above (ANCOVA p = 0.0764, F = 3.56, n = 20). Another example is the proximal width of the tibia, which is broader in Type A than in B for a given trunk length when plotted (Fig. 4f) but ANCOVA finds the difference to be insignificant (ANCOVA p = 0.934, F = 7.22E-3, n = 12; note that the sample size is smaller than in most other cases). In general, the propodial and epipodial elements seem to be better developed in Type A than B for a given trunk length.

Some other quantitative characters are useful for distinguishing between Types A and B only when the sex is first established based on, say, the flipper lengths. For example, the total length of the forelimb and hind limb, respectively, among males is almost constant between Types A and B for a given body trunk length, while females of Type A clearly have longer forelimbs and hind limbs than those of Type B for a given trunk length (Figs 2c and 4d). ANCOVA suggests significant differences in the flipper lengths between females of Types A and B (p = 0.00144, F = 40.1, n = 8 for the forelimb flipper, and p < 0.00241, F = 91.8, n = 6 for the hind limb flipper). However, note that the sample sizes are very small because only females are considered. Similarly, some other features distinguish females of Type B from males of Type B and both sexes of Type A. For example, Type B females have a short radius (Fig. 4a), narrow proximal end of the radius (Fig. 4b), and narrow distal end of the ulna (Fig. 4c) relative to the trunk length, compared to the rest of the sample. These differences are statistically significant except in the first case: ANCOVA results for these regressions all suggest significant differences between Type B females and the rest (p = 0.322, 0.0183, 0.00783, F = 1.06, 7.44, 9.86, and n = 17, 15, 17 in the same order).

### Specimen classification

By combining qualitative and quantitative analyses, 34 specimens were classified into females and males of Types A and B. The result for the morphotype classification is summarized in Table 1. See Supplementary Information for the reasoning of the classification for each specimen. Identities remained ambiguous for the other six specimens, at least to some extent. These specimens are: AGB6253b (probably Type B female but uncertainties remain because the trunk length was estimated); AGB7406 (unidentified; probably Type B because of large size); AGB7405c (Type B of unknown sex); MT10022 (Type B of unknown sex); P45-H85-21 (Type A of unknown sex); P45-H85-23 (Type A of unknown sex). Type B is more abundant than Type A—of the 34 specimens, 13 belongs to Type A and 21 to Type B (Table 2).

**Table 1:** Morphotype classification of the specimens examined.

Museum	Specimen#	Field#	Type	Sex	Museum	Specimen#	Field#	Type	Sex
AGM	AGB6608	A	A	F	AGM	AGB7410	L-1	B	M
	AGB6609	B	A	F		AGB5846a	L-3a	B	M
	AGB7400	CH-502-3	B?	M		AGB5846b	L-3b	B	M
	AGB7401	CH-621-14	B	M		AGB5846c	L-3c	B	?
	AGB6252	CH-628-16	A	M		AGB6607	L-3d	B?	F
	AGB7402	CH-628-17	B	M		AGB6260	L-4	B	M
	AGB7403	CH-628-18	B	M		AGB5855	L-12	A	M
	AGB6259	CH-628-19	A	M		AGB6253	Mother	B	F
	AGB7404	CH-628-20	A	M		AGB6253	BABY	B	F
	AGB6254	CH-628-21	B	F		MT10022	MT10022	B	?
	AGB6255	CH-628-23	B	F		AGB7413	MT10011	A	F
	AGB6262	CH-638-33	A	F		AGB2906	P45-H85-20	A	F
	AGB6605	CH-638-39	B	F		AGB2905	P45-H85-25	A	F
	AGB7406	CHJ-3	?	?	GMPKU	P-1101		B	F
	AGB7407	CHJ-4	B	M		P-3086		B	F
	AGB7408	CHS-1	B	F		P-3093		B	M
	AGB6261	CHS-14	A	F	IVPP	V11361		B	M
	AGB7409	CHS-18	A	M		V11362		A	M
	AGB6256	CHS-3	A	M	NGM	P45-H85-21		A	?
	AGB6258	CHS-5	B	M		P45-H85-23		A	?

**Table 2:** Variations in the trunk length and snout-vent length of different morphotypes.

	Total	TRKL (mm)				SVL (mm)			
	n	mean	min	max	n	mean	min	max	n
Type_A	13	212.1	136.6	317.7	12	300	253	423	7
Type_B	21	279	147.4	469	10	392.8	238.9	578	8
Type_A_F	7	207.7	136.6	261.7	7	290.3	274	309	4
Type_A_M	6	218.4	152	317.7	5	313.5	253	423	3
Type_B_F	8	256.9	147.4	469	6	346.9	238.9	578	4
Type_B_M	13	315.7	196.4	408.1	4	433.9	279	529	4

There are two specimens that were classified as Type B but may represent a third type, namely AGB7400 and AGB6607. These two specimens are unusually immature for their size, i.e., their bones, especially of the limbs, are very poorly ossified although they are as large as subadults to adults of Types A and B, and the intervertebral space remains wide. These specimens possibly represent young individuals of a species that is much larger than what we have at hand. However, the information is too limited at this point to conclude that there was another taxon. Similarly, two specimens that were purposely excluded from the analysis earlier in this paper, i.e., the specimens with ossified centralia^12^, may represent yet another taxon but it is beyond the scope of this study to discuss their taxonomy.

**Figure 5:**
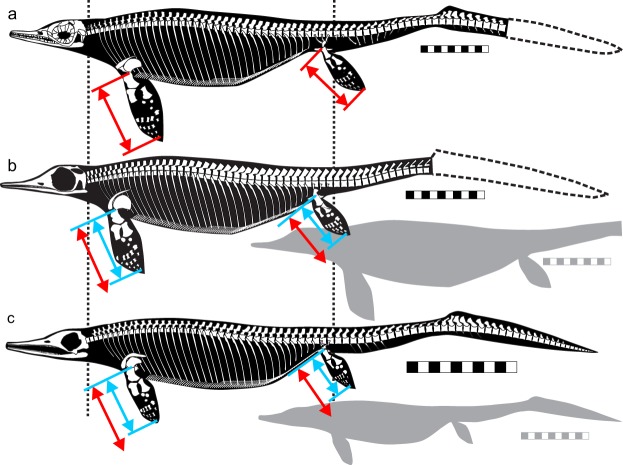
Skeletal reconstruction of morphotypes to show similarities in general body plan and differences in relative flipper lengths. (**a**) The second largest individual of Type B male (AGB6258). (**b**) The largest individual of Type A male (AGB6252). (**c**) The second largest individual of Type A female (AGB6262). Specimens of Type B female are less complete compared to those figured, so no skeletal reconstruction is given for this type. Reconstructions in b and c have been isometrically scaled to have the same trunk length as in a. Many of the differences in relative sizes of features are not readily visible in these reconstructions because many are obscured by allometric growth. Flipper lengths grow nearly isometrically and therefore exhibit visible differences among the morphotypes. Red arrows are the flipper length in a, while those in light blue are for the other two specimens. Qualitative differences are better seen in Fig. 1. Scale bars are 10 cm.

Skeletal reconstructions for typical specimens are given in Fig. 5. None of the specimens of Type B females are as complete as the ones figured, so reconstructions are given only for Type A male and female, and Type B male. All morphotypes are generally similar in body construction, although differences in the relative lengths of flippers (arrows in Fig. 5) are visible in the figure. Many of other differences in relative sizes of features presented above are obscured by allometry and do not stand out in the figure, although they are detected quantitatively, with due consideration of allometry.

## Discussion

As evident from the literature review earlier in this paper, when there is a sexual dimorphism in flipper or limb length in aquatic tetrapods, the flippers/limbs are longer in males than in females for a given body size, without a known counter-example. Moreover, this observation is not coincidental because there is a common cause for the limbs of males to be longer in many species—the flippers/limbs are used as ‘organs of prehension’, sensu Darwin^1,2^, during copulation. Furthermore, no intermediate form between long- and short-flippered individuals is known in the *Chaohusaurus chaoxianensis* complex. Therefore, it is reasonable to recognize the specimens with long flippers as males, and those with short flippers as females.

**Figure 6:**
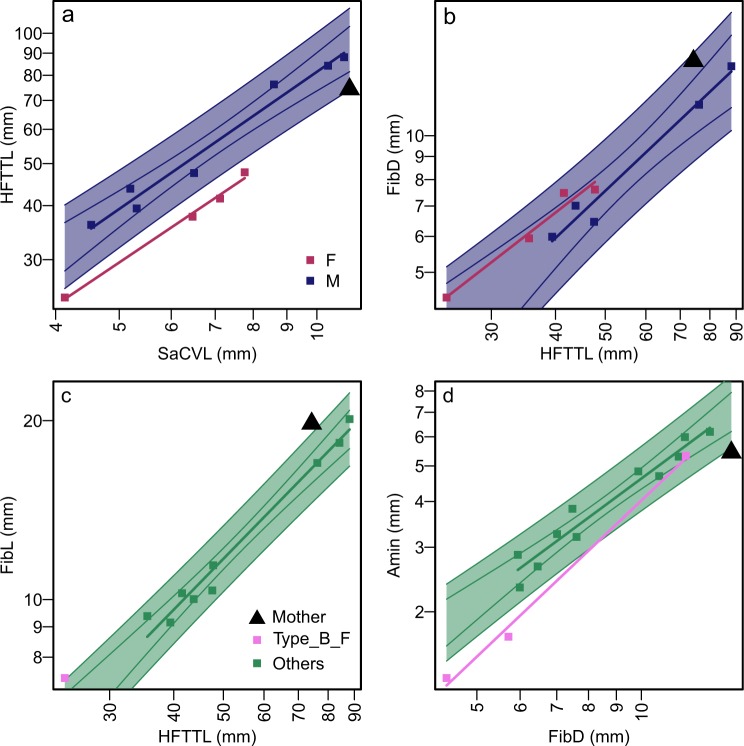
Bone dimensions of pelvic region and classification of gravid specimen (AGB6253). (**a**) HFTTL against SaCVL. (**b**) FibD against HFTTL. (**c**) FibL against HFTTL. (**d**) Amin against FibD. (**a** and **b**) suggest that AGB6253 is female. (**c** and **d**) Suggest that AGB6253 is a female individual of Type B.

This identification can be tested with one specimen for which the sex is known—AGB6253 is undoubtedly female because it has one embryo in the body cavity, and another exiting through the pelvic girdle^38^. Unfortunately, the specimen only preserves the pelvic region of the body, so the trunk length cannot be measured. To facilitate comparisons with other specimens, we were forced to use an inferior proxy for body size, SaCVL (see Materials and Methods). When comparing the hind flipper length to the vertebral length, AGB6253 has a short hind flipper for body size as is the case in other females, although it is placed close to the 95% prediction interval for males (Fig. 6a). We tested this case further by running LDA based on the data for Fig. 6a and using individuals other than AGB6253 for training. Classification by LDA suggested that AGB6253 was female with a posterior probability of 99.7%. Females also have a wider distal end of the fibula for a given hind flipper length than males (Fig. 6b), and LDA based on the data for this figure suggested that AGB6253 was female with a posterior probability of 96.4%.

Two additional pairs of bone dimensions support the identity of AGB6253 as female, thereby revealing features unique to females of Type B. The relative length of the fibula to the entire hind flipper is large in Type B females compared to the other types (Fig. 6c). The data for Fig. 6c only contains one female other than the sample tested, so LDA analysis was not performed. Also, the minimum diameter of the astragalus relative to the distal width of fibula is smaller in Type B females compared to other types (Fig. 6d). LDA based on the data for Fig. 6d suggests that AGB6253 is a female of Type B with a posterior probability of 97.4%.

The female identity of AGB6253 is also supported by multidimensional LDA concatenating the variables mentioned above, namely SaCVL, total length of the hindlimb flipper, minimum diameter of the astragalus, and fibular length and distal width. The posterior probability for AGB6253 being female was 100%, while no specimens in the training data were misclassified by LDA. The same data suggested that AGB6253 was Type B than A, with a posterior probability of 100% and without any misclassification of the training specimens. However, the sample size of the training data was admittedly small (n = 7) given that most specimens lacked at least one of the five variables.

Once the morphotypes are recognized, it is possible to assess size dimorphisms between types. There is a size difference between Types A and B, where Type B is larger than Type A in both the trunk length and SVL on average (Table 2). The differences of the mean values are modestly significant for the trunk length (p = 0.078, F = 3.456) and SVL (p = 0.078, F = 3.63) based on ANOVA of log-transformed metrics—sample sizes are given in Table 2. Then, the observation given above that the propodials and epipodials of Type B are not as well developed as in Type A of the same body length may reflect differences in osteological maturity at a given body size.

Sexual size dimorphism (SSD) within each of Types A and B seems to be present, with males exhibiting larger mean trunk lengths and SVL in both types (Table 2). However, when testing the significance of the differences using ANOVA, they are not statistically significant likely because of the very small sample size for each sex within each type. Larger sample sizes are necessary to scrutinize SSD.

**Figure 7:**
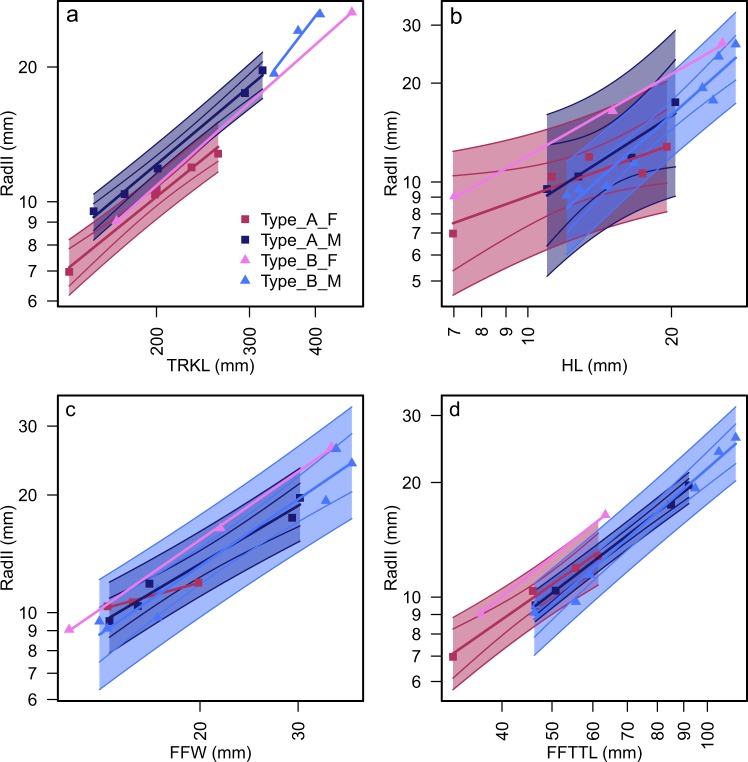
Comparison of carpus length against four measurements. (**a**) RadII against TRKL. (**b**) RadII against HL. (**c**) RadII against FFW. (**d**) RadII against FFTTL. Taxonomic and sexual coherence of the data points seen in (**a**) is not present in (**b**–**d**), suggesting that analyses based on flippers alone may be misleading.

A recent study of the carpal region of *Chaohusaurus* revealed much variation in the length of the carpus relative to humeral length in *C*. *chaoxianensis* complex^12^. The present study shows that this variation does not reflect taxonomy or gender (Fig. 7b). It is true that the carpus is longer in males than in females for a given trunk length (Fig. 7a). However, such patterns disappear when using humeral length, rather than trunk length, to represent size (Fig. 7a vs b). Similarly, the taxon-sex-dependent coherence of the data seen in Fig. 7a disappears when using the total length of the forelimb (Fig. 7c), or the maximum width of the forelimb (Fig. 7d) as the independent variable. This observation suggests that the bone proportions within the flipper may suggest different morphotype groupings than those based on the whole-body data that are preferred. The confusion arising from the bone proportions within the flipper is likely a result of mixing of sexual dimorphism, taxonomic differences, and ontogenetic scaling. It is foreseeable that an analysis based only on the flippers may be misleading in ichthyosaur taxonomy, and caution is required when erecting a taxon based on flippers alone.

Confusions from analyzing only the flippers may result in erroneous interpretation of some features. For example, the carpus is longer relative to the trunk in males than in females (Fig. 7a), likely reflecting the role of the forelimb as an organ of prehension. However, when observing the forelimb alone, a contradictory conclusion may be reached: the carpus is indeed shorter relative to the total forelimb length in males than in females (Fig. 7d). This is because females have shorter flippers for a given body size compared to males, but the carpus is not as shortened as is the rest of the flipper. The retention of the carpus length may suggest that the flexibility of the carpus played an important role in the lifestyle of the *Chaohusaurus chaoxianensis* complex regardless of gender. This reiterates the importance of including a proxy for body size in the data, such as trunk length in this study.

The present study suggests the presence of multiple taxa within the specimen complex resembling *Chaohusaurus chaoxianensis*. Taxa recognized here may represent multiple species. If the two morphotypes are to be recognized as the two species, that would increase the number of *Chaohusaurus* species in the Chaohu Fauna from two to three. These species would be approximately coeval, and at least partially sympatric. The number could become as large as five if the other two minor morphotypes mentioned earlier are recognized as species by future researchers, and this may sound excessive. However, note that *C*. *geishanensis* is very rare at Majiashan, and it is possible that Majiashan was peripheral to the distribution of that species. The same is also true for the two minor morphotypes, leaving the two morphotypes revealed in this manuscript to be the only truly sympatric species. Note also that the rocks bearing *Chaohusaurus* at Majiashan are slope deposits^39^ while *Chaohusaurus* is usually considered inshore animals given the body shape^40^, so it is likely that most of the fossils from the locality may have been derived from animals that lived closer to the coastline than Majiashan. Then, the number of truly sympatric species was probably smaller than it may appear.

Another factor to be considered is the evolutionary rate. It has been suggested that ichthyosauriforms were evolving much faster in the Early Triassic than in later time periods, based on Bayesian phylogenetic framework^41^. Given that the Chaohu fauna represents one of the earliest records of ichthyosauriforms, the high number of observed morphotypes may reflect the unusually high evolutionary rate during the first radiation of Mesozoic marine reptile after the end-Permian mass extinction.

A taxonomic revision of the *Chaohusaurus chaoxianensis* complex would take substantial space, and the descriptive focus of such a paper differs substantially from the analytical focus of the present paper. We therefore defer such a taxonomic revision to a later study.

## Methods

### Specimens

We examined 40 individuals of the *Chaohusaurus chaoxianensis* complex from Majiashan in Chaohu, Anhui Province, China for this study (Table 1). Most specimens were collected through repeated joint excavations that started in 2010. Exceptions include historical specimens described in the previous century^42,43^.

We only included those specimens that are not *C*. *geishanensis*, which is rare and easily identified as mentioned in the Introduction. We also excluded two specimens that resemble *C*. *chaoxianensis* but are unusual for having an exceptionally elongated carpus with ossified centralia—they will be addressed in another study. The sample examined includes the holotype of *C*. *chaoxianensis* (AGB 2905, previously P45-H85-25) and of *C*. *faciles* (AGB 2906; previously P45-H85-20), which has been considered a juvenile of *C*. *chaoxianensis*^8,9^.

Apart from the two holotypes, the following specimens were analyzed: accessioned at the Anhui Geological Museum—AGB 6252, 6253a (mother), 6253b (baby), 6254, 6255, 6256, 6258, 6259, 6260 (Fig. 1a), 6261, 6262, 6264, 6605 (Fig. 1b), 6607, 6608, 6609, 7400, 7401, 7402, 7403, 7404, 7405a,b,c, 7406, 7407, 7408, 7409, 7410, 7411, 7413, and MT10022; accessioned at the Geological Museum of the Peking University—GMPKU P-1101, P-3086, P-3093; accessioned at the Institute of Vertebrate Paleontology and Paleoanthropology—IVPP V11361, V11362; accessioned at Nanjing Geological Museum, Nanjing—P45-H85-21, P45-H85-23. See Table 1 for the field numbers of AGB specimens used in previous publications. AGB stands for Anhui Gushengwu Bowuguan (Anhui Paleontological Museum, the precursor of the Anhui Geological Museum).

The specimens, when known, occurred in a narrow range of fossiliferous rock beds at Majiashan in Chaohu, Hefei City, Anhui Province, China. Our field number for the beds are: 621, 628, 630, 633, 637, and 638, as in previous publications^10,39,41^. Historical specimens lack information on bed numbers but they are most likely from beds 621 or 628—these beds have been the main “ichthyosaur beds” since at least the 1990s, when RM visited Majiashan twice and interviewed the local quarrymen, with help from HY and Junchang Lü. The lithologies of the specimens described by Young^13^ and Chen^42^ match those seen in the general range of beds 621 to 638. The total thickness of beds 621 to 638 is about 12.63 m, representing a span of 0.22 million years according to the astrochronological scale placed on the carbon isotope data^39^.

### Measurements

We used two metrics to represent the body size. The preferred metric is the trunk length (TRKL), which we define as the snout-vent length (SVL) minus the skull length. SVL is measured as the distance between the tip of the snout and the posterior end of the pelvic girdle, along the skull and vertebral column. If the pelvic girdle is not preserved *in situ*, the posterior end of the last sacral vertebra was used instead as the endpoint. We removed the skull length because it was evident that some individuals had larger skulls than the others for the same trunk length. We found the total body length to be unsuitable because of two reasons. The first is sample size: the tail is seldom completely preserved, and the skull often lacks the tip of the snout, limiting the number of specimens for which the total body length could be measured (n = 6). The second is variation. Both skull and tail lengths vary relative to the trunk length within the sample, and both of these two metrics are known to be sexually dimorphic in at least some aquatic tetrapods, as reviewed above. We therefore consider the trunk length to be the best representation of body size for a study involving sexual dimorphism in this specimen complex.

Only about half of the specimens preserve TRKL, while others may have the pelvic region intact without the anterior trunk. For example, the only known definitive female specimen (AGB6253a), with embryos, is preserved in this latter manner^38^. To enable comparison across a wider range of specimens, we performed secondary comparisons using vertebral length as the measure of body size. We used the mean length of the second caudal and second sacral vertebrae (SaCVL) because these two are most frequently measurable in the specimens in question, including AGB6253a. We found that these measurements were more susceptible to errors introduced by fossil deformation than TRKL, and also that their small sizes lead to an increased proportion of measurement errors, so we used these characters only to classify specimens that otherwise cannot be assigned to a morphotype. We regressed TRKL against SaCVL to test the effectiveness of SaCVL in predicting TRKL using Ordinary Least Square regression and calculated 95% confidence and prediction intervals (Fig. 2e,f). As evident from Fig. 2e, the error margin is large as expected, although the mean prediction is an almost isometric relationship between SaCVL and TRKL. When dividing the data according to morphotypes as established later in this paper, there is no morphotype-dependence in this relationship (Fig. 2f), i.e., the use of SaCVL instead of TRKL does not bias our overall conclusion on morphotype membership.

Including the two size proxies, we measured 21 distances of the skull, body trunk, forelimb, hind limb, and tail that may provide information on taxonomy and sexual shape dimorphism. See Table 3 for the list of the measurements and their abbreviations. Measurement values are found in Supplementary Information. Measurements below 174 mm were taken using Mitutoyo digital calipers and recorded to the nearest 0.01 mm. Larger measurements were taken with a narrow metal tape measure, and recorded to the nearest 1 mm. For the baby specimen, TRKL was estimated based on a published reconstruction^38^.

**Table 3:** List of characters and their abbreviations.

Qualitative Traits	
HN	Humeral Notch Shape
NS	Caudalpeak Neural Spine Shape
RF	Radial Flange Shape
Quantitative Traits	
Amin	Astragalus Minimum Diameter
FeL	Femoral Length
FFTTL	Forelimb Flipper Total Length
FFW	Forelimb Flipper Width
FibD	Fibular Distal Width
FibL	Fibular Length
HFTTL	Hindlimb Flipper Total Length
HL	Humeral Length
RadII	Distance between Radius and Metacarpal II
RD	Radius Distal Width
RL	Radius Length
RP	Radius Proximal Width
SKL	Skull Length
SVL	Snout-vent Length
TibII	Distance between Tibia and Metatarsal II
TibL	Tibia Length
TibP	Tibia Proximal Width
TRKL	Trunk Length
UD	Ulnar Distal Width
UL	Ulnar Length
UlV	Distance between Ulna and Metacarpal IV

### Statistical analyses

We tested for the unimodality of our measurements after accounting for body size, based on residuals from a regression line between a given measurement against the trunk length. The ordinary least square regression was used after transforming the data with base-10 logarithm to account for the size dependence of errors in the raw data. P-values for the unimodal distribution, as opposed to multimodal distribution, were calculated according to the Dip-test procedure^44^, as realized in the diptest package of R^45^. The p-values from this test become 1 if the distribution is completely unimodal, and 0 when completely bimodal.

The outcome of the test of unimodality as described above is biased strongly by the regression line used to calculate the residual because it is not unusual for a total data set to have different regression coefficients than when it is divided into appropriate taxonomic or gender groups^46^. The use of the total-data regression coefficients would lead to overestimation of residuals in some samples and underestimation in the others, depending on their position along the x-axis, misleading the conclusion of unimodality (Fig. 2a,b versus c,d). Indeed, an exhaustive trial with the total-data regression suggested that none of the characters was strongly multimodal when multiple morphotypes are recognized through observation. To account for this problem, it is necessary to divide the samples into appropriate taxonomic or gender groups before applying the test of unimodality. We achieved this goal in three steps. We first divided the sample into two morphotypes based on qualitative characters. We then searched for quantitative characters that revealed multimodal distributions of specimens and divided them into ones that recovered the qualitative morphotypes and those that did not. Among the latter, some characters divided the samples congruently with each other, yet differently from the qualitative morphotypes. This second suite of characters was used to divide the morphotypes into two subtypes each.

The differences of the mean values in selected bone dimensions between morphotypes were tested with Analysis of Variance (ANOVA) using the aov function of R^45^. Also, differences in regressions between morphotypes were tested with the Analysis of Covariance (ANCOVA) using the same function. Linear Discriminant Analysis (LDA) was used to test the sex identification of the only gravid female using the lda function of R^45^.

## Electronic supplementary material

Supplementary Information

## Data Availability

The data used for the present study are available in Supplementary Information.
