# Supplementary material for: Separating sexual dimorphism from other morphological variation in a specimen complex of fossil marine reptiles (Reptilia, Ichthyosauriformes, Chaohusaurus)
Source: Sci Rep. 2018 Oct 8;8:14978. doi: 10.1038/s41598-018-33302-4 (PMC6175944; doi:10.1038/s41598-018-33302-4)
Supplement: Supplementary file 1 — Supplementary Information [file 41598_2018_33302_MOESM1_ESM.pdf]

SUPPLEMENTARY INFORMATION for

Separating sexual dimorphism from other morphological variation in a specimen complex of fossil marine reptiles (Reptilia, Ichthyosauriformes, *Chaohusaurus*)

Ryosuke Motani[1*], Jiandong Huang[2*], Da-yong Jiang[3], Andrea Tintori[4], Olivier Rieppel[5], Hailu You[6], Yuan-chao Hu[2], Rong Zhang[2]

[1] Department of Earth and Planetary Sciences, University of California, Davis, CA 95616-8605, U. S. A., rmotani@ucdavis.edu;

[2] Department of Research, Anhui Geological Museum, Jiahe Road 999, Hefei, Anhui 230031, People's Republic of China, jiandongh2008@163.com;

[3] Laboratory of Orogenic Belt and Crustal Evolution, Ministry of Education; Department of Geology and Geological Museum, Peking University, Beijing 100871, People's Republic of China, djiang@pku.edu.cn;

[4] Dipartimento di Scienze della Terra, Università degli Studi di Milano, Via Mangiagalli 34-20133 Milano, andrea.tintori@unimi.it;

[5]Integrative Research Center, The Field Museum, Chicago, IL 60605-2496, U. S. A., orieppel@fieldmuseum.org;

[6] Institute of Vertebrate Paleontology and Paleoanthropology, Chinese Academy of Science, 142 Xizhimenwai Street, 100044 Beijing, China, youhailu@ivpp.ac.cn.

[*]Correspondence to rmotani@ucdavis.edu or jiandongh2008@163.com

Supplementary Table S1. Morphotype identifications and features used.

Museum	Specimen#	Field #	Morphotype	Sex		Features used for morphotype identification and individual results														
					Feature2	—	—	—	TRKL									SKL		HFTTL
					Feature1	NS	RF	HN	FFTTL	HFTTL	RadII	FFW	RP	UL	UD	FeL	TibP	FFTTL	RL	FibL2
AGM	AGB6608	A	Type A	F		A	A	A	—	A	—	—	—	—	—	—	—	—	A	—
	AGB6609	B	Type A	F		A	—	—	—	—	—	—	—	—	—	—	—	—	A	—
	AGB7400	CH-502-3	Type B?	M		—	B	B	—	—	—	—	—	—	—	B	B	—	—	—
	AGB7401	CH-621-14	Type B	M		B	B	B	—	—	—	—	—	—	—	—	B	—	—	—
	AGB6252	CH-628-16	Type A	M		—	A	—	—	—	—	—	—	—	—	—	A	—	—	—
	AGB7402	CH-628-17	Type B	M		B	—	—	—	—	—	—	—	—	—	—	—	—	—	—
	AGB7403	CH-628-18	Type B	M		—	B	—	—	—	—	—	—	—	—	—	—	—	—	—
	AGB6259	CH-628-19	Type A	M		—	A	A	—	—	—	—	—	—	—	—	A	—	—	—
	AGB7404	CH-628-20	Type A	M		A	—	—	—	—	—	—	—	—	—	—	—	—	—	—
	AGB6254	CH-628-21	Type B	F		—	—	—	B	—	—	B	—	—	—	—	—	B	—	—
	AGB6255	CH-628-23	Type B	F		—	B	B	—	—	—	—	—	—	—	—	—	B	—	—
	AGB6262	CH-638-33	Type A	F		A	A	A	A	A	—	—	—	—	—	—	A	—	A	—
	AGB6605	CH-638-39	Type B	F		—	B	B	—	—	—	B	—	—	—	—	—	—	—	—
	AGB7406	CHJ-3	?	?		—	—	— —	—	—	—	—	—	—	—	—	—	—	—	—
	AGB7407	CHJ-4	Type B	M		—	B	—	—	—	—	—	—	—	—	—	—	—	—	—
	AGB7408	CHS-1	Type B	F		—	—	B	—	—	—	B	—	B	B	B	—	—	—	—
	AGB6261	CHS-14	Type A	F		—	—	A	—	—	—	—	—	—	—	—	—	—	—	—
	AGB7409	CHS-18	Type A	M		A	A	A	—	—	—	—	—	—	—	—	A	—	—	—
	AGB6256	CHS-3	Type A	M		A	A	A	—	—	—	—	—	—	—	—	A	—	—	—
	AGB6258	CHS-5	Type B	M		B	B	B	—	—	—	—	—	—	—	—	B	—	—	—
	AGB7410	L-1	Type B	M		—	B	B	—	—	—	—	—	—	—	—	—	—	—	—
	AGB5846a	L-3a	Type B	M		B	B	—	—	—	—	—	—	—	—	—	—	—	—	—
	AGB5846b	L-3b	Type B	M		—	B	B	—	—	—	—	—	—	—	—	—	—	—	—
	AGB5846c	L-3c	Type B	?		B	—	—	—	—	—	—	—	—	—	—	—	—	—	—
	AGB6607	L-3d	Type B?	F		—	B	B	B	B	—	—	—	—	—	—	—	B	—	—
	AGB6260	L-4	Type B	M		B	B	B	—	—	—	—	—	—	—	—	—	—	—	—
	AGB5855	L-12	Type A	M		A	A	A	—	—	—	—	—	—	—	—	—	—	—	—
	AGB6253	Mother	Type B	F		—	—	—	—	—	—	—	—	—	—	—	—	—	—	B
	AGB6253	BABY	Type B	F		—	—	—	—	—	—	—	—	B	—	—	—	—	—	—
	MT10022	MT10022	Type B	?		B	B	B	—	—	—	—	—	—	—	—	—	—	—	—
	AGB7413	MT10011	Type A	F		—	—	—	A	A	A	—	—	—	—	—	A	—	A	—
	AGB2906	P45-H85-20	Type A	F		—	A	A	A	—	A	—	—	—	—	—	—	—	A	—
	AGB2905	P45-H85-25	Type A	F		A	A	A	A	A	A	—	—	—	—	—	A	—	—	—
GMPKU	P-1101		Type B	F		—	B	B	B	B	—	—	—	—	—	B	B	B	—	B
	P-3086		Type B	F		B	B	—	—	—	—	B	B	—	—	B	B	—	—	—
	P-3093		Type B	M		—	B	B	—	—	—	—	—	—	—	—	—	—	—	—
IVPP	V11361		Type B	M		—	B	B	—	—	—	—	—	—	—	—	—	—	—	—
	V11362		Type A	M		—	A	A	—	—	—	—	—	—	—	—	—	—	—	—
NGM	P45-H85-21		Type A	?		A	—	—	—	—	—	—	—	—	—	—	—	—	—	—
	P45-H85-23		Type A	?		A	—	—	—	—	—	—	—	—	—	—	—	—	—	—

Supplementary Table S2. Sex identifications and features used.

Museum	Specimen#	Field #	Morphotype	Sex		Features used for sex identification and individual results																				
					Feature2	TRKL									SaCVL				SKL		FFTTL			HFTTL		UL
					Feature1	FFTTL	HFTTL	SKL	RadII	FFW	RP	UL	UD	FeL	HTTL2	TibL	FibL	TibII	FFTTL	RL	RadII	UlV	HL	TibP2	FibL2	RP
AGM	AGB6608	A	Type A	F		—	F	—	F	—	—	—	—	—	F	—	—	—	—	F	—	—	—	—	—	—
	AGB6609	B	Type A	F		F	—	F	F	—	—	—	—	—	—	—	—	—	—	F	—	F	—	—	—	—
	AGB7400	CH-502-3	Type B?	M		—	—	M	—	—	—	—	—	—	—	—	—	—	—	—	—	—	—	—	—	—
	AGB7401	CH-621-14	Type B	M		M	—	M	M	—	—	—	—	—	—	—	—	—	—	—	M	M	M	—	—	—
	AGB6252	CH-628-16	Type A	M		M	—	M	M	—	—	—	—	—	—	—	—	—	—	—	—	M		—	—	—
	AGB7402	CH-628-17	Type B	M		—	—	—	—	—	—	—	—	—	M	—	—	—	—	—	—	—	—	—	—	—
	AGB7403	CH-628-18	Type B	M		—	—	—	—	—	—	—	—	—	—	—	—	—	—	—	—	—	—	—	—	M
	AGB6259	CH-628-19	Type A	M		M	—	—	M	—	—	—	—	—	—	—	—	—	—	—	—	M	—	—	—	—
	AGB7404	CH-628-20	Type A	M		—	—	—	—	—	—	—	—	—	—	M	M	M	—	—	—	—	—	—	—	—
	AGB6254	CH-628-21	Type B	F		F	—	F	—	F	—	—	—	—	—	—	—	—	F	—	—	—	—	—	—	—
	AGB6255	CH-628-23	Type B	F		—	—		—	—	—	—	—	—	—	—	—	—	F	—	F	F	F	—	—	—
	AGB6262	CH-638-33	Type A	F		F	F	F	—	—	—	—	—	—	—	—	—	—	—	F	—	—	—	F	—	—
	AGB6605	CH-638-39	Type B	F		—	—	F	—	F	—	—	—	—	—	—	—	—	—	—	—	—	—	—	—	—
	AGB7406	CHJ-3	?	?		—	—	—	—	—	—	—	—	—	—	—	—	—	—	—	—	—	—	—	—	—
	AGB7407	CHJ-4	Type B	M		—	—	—	—	—	—	—	—	—	—	—	—	—	—	—	—	—	M	—	—	—
	AGB7408	CHS-1	Type B	F		—	—	F	F	F	—	F	F	F	—	—	—	—	—	—	—	—	—	—	—	—
	AGB6261	CHS-14	Type A	F		—	—	F	—	—	—	—	—	—	—	—	—	—	—	—	—	—	—	—	—	—
	AGB7409	CHS-18	Type A	M		—	—	M	M	—	—	—	—	—	M	—	—	—	—	—	—	M	—	M	—	—
	AGB6256	CHS-3	Type A	M		—	—	M	M	—	—	—	—	—	M	—	—	—	—	—	—	—	—	M	—	—
	AGB6258	CHS-5	Type B	M		M	M	M	M	—	—	—	—	—	M	—	—	—	—	—	M	M	M	M	—	—
	AGB7410	L-1	Type B	M		—	—	—	—	—	—	—	—	—	—	—	—	—	—	—	—	—	—	—	—	—
	AGB5846a	L-3a	Type B	M		—	—	—	—	—	—	—	—	—	—	—	—	—	—	—	M	M	M	M	—	—
	AGB5846b	L-3b	Type B	M		—	—	—	—	—	—	—	—	—	—	—	—	—	—	—	M	M	M	—	—	—
	AGB5846c	L-3c	Type B	?		—	—	—	—	—	—	—	—	—	—	—	—	—	—	—	—	—	—	—	—	—
	AGB6607	L-3d	Type B?	F		F	F	F	—	—	—	—	—	—	—	—	—	—	F	—	—	—	F	—	—	—
	AGB6260	L-4	Type B	M		M	M	M	M	—	—	—	—	—	M	—	—	—	—	—	M	—	M	—	—	—
	AGB5855	L-12	Type A	M		M	M	—	M	—	—	—	—	—	M	—	—	—	—	—	—	M	—	—	—	—
	AGB6253	Mother	Type B	F		—	—	—	—	—	—	—	—	—	F	—	—	—	—	—	—	—	—	F	F	—
	AGB6253	BABY	Type B	F		—	—	F	—	—	—	F	—	—	—	—	—	—	—	—	—	—	—	—	—	—
	MT10022	MT10022	Type B	?		—	—	—	—	—	—	—	—	—	—	—	—	—	—	—	—	—	—	—	—	—
	AGB7413	MT10011	Type A	F		F	F	F	F	—	—	—	—	—	—	—	—	—	—	F	—	F	F	F	—	—
	AGB2906	P45-H85-20	Type A	F		F	—	F	F	—	—	—	—	—	—	—	—	—	—	F	—	—	—	—	—	—
	AGB2905	P45-H85-25	Type A	F		F	F		F	—	—	—	—	—	F	—	—	—	—	—	—	—	—	F	—	—
GMPKU	P-1101		Type B	F		F	F	F	F	—	F	F	F	F	F	—	—	—	F	—	F	—	—	—	F	—
	P-3086		Type B	F		—	—	—	—	F	F	—	—	F	—	—	—	—	—	—	—	—	—	—	—	—
	P-3093		Type B	M		—	—	—	—	—	—	—	—	—	—	—	—	—	—	—	—	—	M	—	—	—
IVPP	V11361		Type B	M		—	—	—	—	—	—	—	—	—	—	—	—	—	—	—	M	—	M	—	—	—
	V11362		Type A	M		—	—	—	—	—	—	—	—	—	—	—	—	—	—	—	—	—	—	—	—	—
NGM	P45-H85-21		Type A	?		—	—	—	—	—	—	—	—	—	—	—	—	—	—	—	—	—	—	—	—	—
	P45-H85-23		Type A	?		—	—	—	—	—	—	—	—	—	—	—	—	—	—	—	—	—	—	—	—	—

Supplementary Table S3. Measurements used in mm.

Museum	Specimen #	Field #	Amin	FeL	FFTTL	FFW	FibD	FibL	HFTTL	HL	RadII	RD	RL	RP	SKL	SVL	TibII	TibL	TibP	TRKL	UD	UL	UlV
AGM	AGB6608	A				15.15			37.7	17.38	10.63	6.96	14.54	9.5	74.81	275	4.68			200.19	8.25	13.68	6.42
	AGB6609	B			55.64	19.87				13.44	11.93		13.65		75.97	309				233.03	9.82	12.69	9.96
	AGB7400	CH-502-3		6.96			5.88	7.85		10.54					82.62	279		7.57	3.62	196.38			
	AGB7401	CH-621-14	5.99		94.7	33.66	12.01	19.13		23.22	19.32	10.56	21.69	14.71	108	442		17.99	6.98	334	15.98	20.52	15.04
	AGB6252	CH-628-16	4.83	14.59	92.03	30.23	9.87				19.66	8.25	19.7	13.18	105.32	423		13.67	7.33	317.68	14.86	19.17	13.11
	AGB7402	CH-628-17	5.3	16.59			11.7	16.98	76.2									15.31					
	AGB7403	CH-628-18									18.389		16.697	10.594	93.266						11.26	16.303	11.954
	AGB6259	CH-628-19	4.69	14.93	85.03	29.27	10.78	15.84		20.38	17.49	9.03	18.35	13.34			10.54	14.38	7.28	294.13	13.84	17.46	11.96
	AGB7404	CH-628-20	6.193	17.81			13.35	18.792									12.466	18.686	7.949				
	AGB6254	CH-628-21			28.62	8.61									67.02					147.4			
	AGB6255	CH-628-23	1.71		63.43	21.77	5.71	8.11		15.06	16.44	7.91	14.75	10.6	100.42			7.31	4.46		10.53	13.46	13.37
	AGB6262	CH-638-33	3.82	8.68	53.52	16.67	7.48	10.25	41.53	14.03		7.21	13.94	9.18	75.62	305		9.61	5.38	229.38	9.51	14.03	
	AGB6605	CH-638-39				12.09				11.02					66.29	245				181			
	AGB7407	CHJ-4		16.08	92.48	35.38	12.59	18.165		24.93			23.05	17		484		18.39	8.93		15.01	24	
	AGB7408	CHS-1		20.39		34.42				25.59	26.5	11.56	20.96		108.96	578				469.04	11.71	22.31	17.05
	AGB6261	CHS-14								16.98					81.364					219.18			
	AGB7409	CHS-18	2.33	8.72	50.84	15.47	5.99	9.15	39.4	12.76	10.4	6.05	11.57	8.49	79.03	253.03	6.72	9.3	4.38	174	9.2	12.13	8.06
	AGB6256	CHS-3	3.26	9.43		16.23	7.01	10.02	43.76	16.5	11.86	5.47	13.5	9.38	87	288	7.15	9.87	4.36	201	7.35	12.46	9.26
	AGB6258	CHS-5	5.19	16.9	105.22	37.46		18.35	84.18	25.11	24.09	11.68	22.88	16.19	115.91	486.91	18.11	17.18	7.56	371	15.92	20.04	20.01
	AGB5846a	L-3a			46.22	13.63				12.06	9.11	4.36	10.28	6.7	80.61						8.25	9.55	6.76
	AGB6607	L-3d			64.93				44.9	13.71					98.82	428				329.18			
	AGB7410	L-1								24.46	17.71		22.39	13.91							14.99	23.5	
	AGB5855	L-12		7.95	46.46	13.76			36.12	10.97	9.52	4.81	9.74	5.69			5.53			152	6.36	9.04	7.38
	AGB5846b	L-3b	2.66	9.84	55.64	16.78	6.46	10.36	47.56	14.6	9.72	4.89	13.34	7.54				9.39	4.42		7.69	11.83	6.77
	AGB6260	L-4		19.38	113.46	35.1	14.22	20.12	88.08	27.27	26.23	11.94	24.02	13.34	120.9	529				408.1	11.88	24.79	
	AGB6253	Mother	5.44	18.08			14.61	19.78	74.3								14.9	16.91	8.92				
	AGB7413	MT10011	2.86	8.17	45.9	13.65	5.94	9.38	35.52	11.22	10.38	4.36	11.84	6.93	75.36	274	6.12	8.24	4.59	198.64	8.12	10.16	7.64
	AGB2906	P45-H85-20			32.16					6.97	6.97	2.64	6.64	4.54	58.97					136.6	4.77	6.87	
	AGB2905	P45-H85-25	3.2	11.9	61.47		7.61	11.42	47.74	19.56	12.8	6.84	15.53	10.42			6.1	10.14	5.5	261.7	8.36	15.38	
GMPKU	P-1101		1.32	5.22	36.35	11.65	4.4	7.38	24.53	7.02	9.05	3.42	7.74	4.76	70.86	238.86	2.5	6.01	3.42	168	3.68	7.94	
	P-3086		5.33	17.195			12.06	18.005		18.7		8.03	16.32	11.42				15.925	7.06	415.3			
	P-3093				37.87					10.42		4.24	10.2	5.98								9.57	
IVPP	V11361				59.3	16.8				16.71	11.44	6.61	15.93	9.84							8.32	15.06	
	V11362				46.5	13.2				12.8	9.5	5.33	12.12	7.73							8.08	11.09	
